# Genome Sequence of *Paracoccus contaminans* LMG 29738^T^, Isolated from a Water Microcosm

**DOI:** 10.1128/genomeA.00487-17

**Published:** 2017-06-08

**Authors:** Philipp Aurass, Susanne Karste, Eva Trost, Stefanie P. Glaeser, Peter Kämpfer, Antje Flieger

**Affiliations:** a Division of Enteropathogenic Bacteria and Legionella (FG11), Robert Koch-Institut, Wernigerode, Germany; b Institut für Angewandte Mikrobiologie, Universität Giessen, Giessen, Germany

## Abstract

We announce here the complete genome sequence of *Paracoccus contaminans* LMG 29738^T^, which we recently isolated from a contaminated water microcosm. The genome consists of a 2.94-Mb chromosome and a 94-kb plasmid. To our knowledge, we provide the first DNA methylation analysis of a *Paracoccus* species.

## GENOME ANNOUNCEMENT

The genus *Paracoccus* represents coccoid or short-rod Gram-negative bacteria and currently constitutes more than 50 named species. Among them, two species have been of special research interest. *Paracoccus yeei* has been repeatedly implicated in disease and is an untypical opportunistic pathogen (1, 2). *Paracoccus denitrificans* constitutes the best-studied member of the genus *Paracoccus* and shows versatile metabolic traits. An important property of this strain is its capability to denitrify and to degrade *N*ʹ*N*ʹ-dimethylamine (3–5). We recently isolated and phenotypically characterized a novel *Paracoccus* species, termed *Paracoccus contaminans* (6). Here, we announce the genome sequence of *P. contaminans* LMG 29738^T^, including the first base modification analysis of a *Paracoccus* species.

Genome sequencing was performed on a PacBio RSII system using two single-molecule real-time (SMRT) cells to generate an average reference coverage of 278-fold. Genomic DNA was isolated using the QIAamp kit (Qiagen). Library preparation, SMRT sequencing, genome assembly using an HGAP-based pipeline, and all subsequent bioinformatics, including contig circularization and base modification analysis, were performed by GATC biotech (Hinden, Germany). Two contigs were found to represent circular sequences corresponding to a 2.94-Mb chromosome (base coverage, 343-fold) and a 95-kb plasmid, which we termed pPC1 (base coverage, 113-fold). The *P. contaminans* LMG 29738^T^ chromosome has an average G+C content of 68.6%, and the mean G+C content of pPC1 is slightly higher, at 69.6%. Gene identification and annotation of the genome were performed by the Rapid Annotations using Subsystems Technology (RAST) server (7) and the NCBI Prokaryotic Genome Annotation Pipeline (PGAP).

Based on the RAST results, 2,854 coding sequences (CDSs) were detected on the chromosome, and 89 CDSs were detected on the plasmid. Moreover, 6 ribosomal RNAs and 46 tRNAs were identified. A total of 2,097 of the chromosomal CDSs and 78 of the plasmid CDSs were assigned putative functions. Annotation via RAST revealed features consistent with other available *Paracoccus* genomes but also showed distinguishing contents compared with the two available complete *Paracoccus* chromosomes from *P. denitrificans* and *P. aminophilus*. No genes involved in denitrification were identified. In agreement with its observed motility (6), 49 genes were implicated in flagellar motility, which is in contrast to nonmotile *P. denitrificans* and *P. aminophilus* (6 and 15 flagellar motility-related genes, respectively). Moreover, *P. contaminans* is equipped with genes involved in l-fucose utilization (those encoding l-fuconolactone-hydrolase, FucD, FucD2, 2-keto-3-deoxy-l-fuconate-dehydrogenase, and 2,4-diketo-3-deoxy-l-fuconate-dehydrogenase), a putative AttEFGH ABC transport system, and five putative clustered regularly interspaced short palindromic repeat (CRISPR)-associated (Cas) proteins (Cas1, Cas2, Cas3, Csd1, and Cas5d). Analysis of the *P. contaminans* chromosome by means of antiSMASH (8) revealed the presence of four clusters for secondary metabolite generation, specifically, two microcin clusters, a lassopeptide cluster, and a homoserinlactone cluster.

### Accession number(s).

Sequences and base modification data have been deposited in GenBank under the accession numbers CP020612 (chromosome) and CP020613 (pPC1).
